# Supplementary material for: Maternal and Neonatal Factors Modulating Breast Milk Cytokines in the First Month of Lactation
Source: Antioxidants (Basel). 2023 Apr 25;12(5):996. doi: 10.3390/antiox12050996 (PMC10215605; doi:10.3390/antiox12050996)
Supplement: Supplementary file 1 [file antioxidants-12-00996-s001.zip › antioxidants-2305654-supplementary.pdf]

Table S1. Breast milk cytokine levels and oxidative status at 7 and 28 days of lactation from women with neonates without adverse outcomes separated by neonatal sex.

	**Day 7**			**Day 28**		
**Cytokines and Oxidative status**	**Male (*n* =15)**	**Female (*n* =24)**	***p***	**Male (*n* =14)**	**Female (*n* =18)**	***p***
IL-10 Ln(pg/mL)	-0.20 [-0.22; -0.13]	-0.18 [-0.20; -0.07]	0.39	-0.17 [-0.22; -0.13]	-0.17 [-0.21; -0.07]	0.76
IL-13 Ln(pg/mL)	-0.51 [-0.55; -0.40]	-0.50 [-0.53; -0.42]	0.79	0.76 [0.13; 1.00]	0.59 [-0.13; 0.82]	0.49
IL-8 Ln(pg/mL)	-0.22 [-0.49; 1.74]	-0.22 [-0.37; 0.69]	0.97	-0.41 [-0.50; -0.22]	-0.58 [-0.63; -0.24]	0.40
MCP-1 Ln(pg/mL)	0.27 [-0.41; 1.04]	0.95 [0.07; 1.32]	0.11	-0.79 [-0.93; -0.17]	-0.93 [-0.97; -0.55]	0.17
TNFα Ln(pg/mL)	-0.17 [-0.38; 0.10]	-0.20 [-0.30; 0.12]	0.51	-0.27 [-0.34; -0.08]	-0.32 [-0.40; -0.26]	0.27
Inflammatory index	0.01 [-0.25; 0.47]	-0.12 [-0.39; 0.73]	0.76	-0.27 [-0.34; -0.08]	-0.13 [-0.40; 0.21]	0.91
ABTS Ln(mg TE/mL)	1.02 [0.62; 1.39]	0.40 [-0.01; 0.90]	0.004	-0.72 [-1.14; -0.44]	-1.04 [-1.24; -0.55]	0.44
MDA+HNE Ln(μM)	-0.09 [-0.32; 0.53]	0.17 [-0.19; 0.59]	0.50	-0.56 [-0.94; -0.06]	-0.45 [-0.96; -0.03]	0.91

Data show median and interquartile range [Q1; Q3] of cytokines after logarithm (Ln) transformation and typification. The *p*-Value (P) was extracted from independent Wilcoxon test. ABTS: 2,20-azino-bis-3-ethylbenzothiazoline-6-sulfonic acid; TE: Trolox equivalent; MDA: malondialdehyde; HNE: 4-Hydroxy-Trans-2-Nonenal.

Table S2. Breast milk cytokine levels and oxidative status at 7 and 28 days of lactation from women with and without C-section.

	Day 7			Day 28		
**Cytokine and Oxidative status**	**Vaginal (*n* =30)**	**C-section (*n* =14)**	***p***	**Vaginal (*n* =24)**	**C-section (*n* =12)**	***p***
IL-10 Ln(pg/mL)	-0.19 [-0.21; -0.13]	-0.17 [-0.20; -0.02]	0.20	-0.16 [-0.21; -0.06]	-0.19 [-0.22; -0.16]	0.26
IL-13 Ln(pg/mL)	-0.50 [-0.53; -0.42]	-0.52 [-0.55; -0.42]	0.47	0.66 [0.10; 0.99]	0.61 [-0.02; 0.78]	0.66
IL-8 Ln(pg/mL)	-0.22 [-0.48; 0.73]	0.09 [-0.35; 1.37]	0.44	-0.42 [-0.62; -0.20]	-0.49 [-0.61; -0.32]	0.68
MCP-1 Ln(pg/mL)	0.28 [-0.25; 1.16]	1.18 [0.71; 1.63]	0.018	-0.92 [-0.97; -0.60]	-0.48 [-0.92; -0.23]	0.17
TNFα Ln(pg/mL)	-0.19 [-0.37; 0.03]	-0.19 [-0.26; 0.24]	0.29	-0.28 [-0.38; -0.08]	-0.32 [-0.40; -0.27]	0.37
Inflammatory index	-0.11 [-0.28; 0.62]	-0.08 [-0.38; 0.42]	0.99	-0.13 [-0.44; 0.24]	-0.30 [-0.38; 0.26]	0.83
ABTS Ln(mg TE/mL)	0.69 [0.01; 1.12]	0.87 [0.51; 1.10]	0.29	-0.92 [-1.24; -0.53]	-0.79 [-1.15; -0.36]	0.50
MDA+HNE Ln(μM)	0.05 [-0.23; 1.03]	0.00 [-0.63; 0.24]	0.26	-0.41 [-1.00; -0.13]	-0.60 [-0.92; 0.06]	0.76

Data show median and interquartile range [Q1; Q3] of cytokines after logarithm (Ln) transformation and typification. Women c-section, cesarean section. The *p*-Value (P) was extracted from independent Wilcoxon test. ABTS: 2,20-azino-bis-3-ethylbenzothiazoline-6-sulfonic acid; TE: Trolox equivalent; MDA: malondialdehyde; HNE: 4-Hydroxy-Trans-2-Nonenal.

Table S3. Breast milk cytokine levels and oxidative status at 7 and 28 days of lactation comparing women with and without obstetric complications.

	Day 7			Day 28		
**Cytokine and Oxidative status**	**No (*n* = 27)**	**Yes (*n* = 12)**	***p***	**No (*n* = 25)**	**Yes (*n* =11)**	***p***
IL-10 Ln(pg/mL)	-0.20 [-0.21; -0.09]	-0.15 [-0.20; -0.07]	0.15	-0.18 [-0.22; -0.07]	-0.16 [-0.18; -0.10]	0.31
IL-13 Ln(pg/mL)	-0.52 [-0.54; -0.48]	-0.46 [-0.51; -0.29]	0.10	0.60 [-0.23; 0.84]	0.61 [0.41; 1.01]	0.43
IL-8 Ln(pg/mL)	-0.22 [-0.40; 0.66]	0.24 [-0.37; 2.77]	0.25	-0.48 [-0.63; -0.27]	-0.42 [-0.55; -0.21]	0.54
MCP-1 Ln(pg/mL)	0.46 [-0.12; 1.26]	0.95 [0.19; 1.21]	0.59	-0.86 [-0.96; -0.54]	-0.63 [-0.94; -0.28]	0.60
TNFα Ln(pg/mL)	-0.25 [-0.35; 0.00]	-0.04 [-0.19; 0.38]	0.05	-0.33 [-0.40; -0.19]	-0.27 [-0.33; -0.26]	0.71
Inflammatory index	-0.08 [-0.38; 0.99]	-0.18 [-0.67; 0.04]	0.33	0.07 [-0.38; 0.35]	-0.39 [-0.77; -0.11]	0.021
ABTS Ln(mg TE/mL)	0.87 [0.16; 1.09]	0.69 [0.18; 1.05]	0.81	-0.91 [-1.36; -0.49]	-0.98 [-1.14; -0.49]	0.73
MDA+HNE Ln(µM)	0.04 [-0.35; 0.56]	0.13 [-0.07; 0.75]	0.56	-0.50 [-1.06; 0.06]	-0.36 [-0.73; -0.17]	0.43

Data show median and interquartile range [Q1; Q3] of cytokines after logarithm (Ln) transformation and typification. Women diagnosed with an obstetric complication were categorized as “yes”, otherwise they were categorized as "no". The *p*-Value (P) was extracted from independent Wilcoxon test. ABTS: 2,20-azino-bis-3-ethylbenzothiazoline-6-sulfonic acid; TE: Trolox equivalent; MDA: malondialdehyde; HNE: 4-Hydroxy-Trans-2-Nonenal.
